# Impact of the COVID-19 Pandemic and Public Restrictions on Outcomes After Catheter Ablation of Atrial Fibrillation

**DOI:** 10.3389/fcvm.2022.836288

**Published:** 2022-03-24

**Authors:** Daehoon Kim, Hee Tae Yu, Tae-Hoon Kim, Jae-Sun Uhm, Boyoung Joung, Moon-Hyoung Lee, Hui-Nam Pak

**Affiliations:** Yonsei University College of Medicine, Yonsei University Health System, Seoul, South Korea

**Keywords:** atrial fibrillation, COVID-19, pandemic, catheter ablation, rhythm outcome

## Abstract

**Background:**

Here we aimed to analyze changes in the outcomes of atrial fibrillation (AF) catheter ablation (AFCA) during the coronavirus disease 2019 (COVID-19) pandemic and examine the relationship between rhythm outcomes and the stringency of government social distancing measures.

**Methods:**

We included 453 patients who underwent *de novo* AFCA between May 2018 and October 2019 (pre-COVID-19 era) and 601 between November 2019 and April 2021 (COVID-19 era). The primary outcome was late recurrence, defined as any episode of AF or atrial tachycardia documented after a 3-month blanking period. A multivariable Cox regression analysis was performed to estimate the relative hazards of AF recurrence in the two eras.

**Results:**

In the study population (24.3% women; median age, 60 years), 660 (62.6%) patients had paroxysmal AF. Among those with paroxysmal AF, the late recurrence rate was significantly lower in the COVID-19 era than in the pre-COVID-19 era [9.4% vs. 17.0%, respectively, log-rank *P* = 0.004; adjusted hazard ratio (HR) 0.56, 95% confidence interval (CI) 0.35–0.90] during a median follow-up of 11 months. In patients with persistent AF, the late recurrence rate did not significantly differ between the pre-COVID-19 and COVID-19 era groups (18.9% vs. 21.5%, respectively; log-rank *P* = 0.523; adjusted HR 0.84, 95% CI 0.47–1.53) during the median follow-up of 11 months.

**Conclusion:**

A decrease in AF recurrence after catheter ablation was observed in patients with paroxysmal AF during the COVID-19 outbreak, whereas no change was observed in those with persistent AF.

## Introduction

Severe acute respiratory syndrome coronavirus-2, which causes coronavirus disease 2019 (COVID-19), has affected over 2 million people worldwide (1). As a result, many countries have implemented public health restrictions to mitigate its spread. In Korea, non-pharmaceutical interventions (NPIs), including compulsory mask-wearing, social distancing, and enhanced screening and testing, were implemented in February 2020, the early phase of the outbreak (Supplementary Figure 1A) (2).

Electrophysiologic issues, including arrhythmias or device-related issues, have been increasingly recognized as a manifestation of COVID-19. While the need for services from electrophysiology laboratories continues to increase, a recent consensus paper recommended canceling or postponing elective cases during the pandemic (3). However, little is known about the impact of the COVID-19 pandemic and associated public health restrictions on clinical outcomes of catheter ablation for atrial fibrillation (AF).

## Methods

### Study Population

This single-center retrospective observational study aimed to analyze changes in the outcomes of AF catheter ablation (AFCA) during the COVID-19 pandemic and examine the relationship between rhythm outcomes and the stringency of government social distancing measures. We included 453 consecutive patients who underwent *de novo* AFCA between May 2018 and October 2019 (18 months of the pre-COVID-19 era) and 601 between November 2019 and April 2021 (18 months of the COVID-19 era) at Severance Cardiovascular Hospital, a tertiary referral center in the Republic of Korea (Supplementary Figures 1B,C). The study protocol adhered to the Declaration of Helsinki and was approved by our institutional review board. Written informed consent was obtained from all patients (ClinicalTrials.gov: NCT02138695). The exclusion criteria were: (1) permanent AF refractory to electrical cardioversion; (2) AF with valvular disease ≥ grade 2; (3) a previous cardiac surgery with concomitant AF surgery or AFCA; and (4) empirical extra-pulmonary vein (PV) ablations other than the typical circumferential PV isolation. All antiarrhythmic drugs (AADs) were discontinued for at least five half-lives, and amiodarone was stopped at least 4 weeks before the procedure.

### Echocardiographic Evaluation

All patients underwent transthoracic echocardiography (Sonos 5500, Philips Medical System, Andover, MA or Vivid 7, GE Vingmed Ultrasound, Horten, Norway) prior to their ablation. Chamber size, left ventricular ejection fraction, transmitral Doppler flow velocity, and the ratio of early diastolic peak mitral inflow velocity to early diastolic mitral annular velocity (E/Em) were acquired according to the American Society of Echocardiography guidelines (4).

### Electrophysiological Mapping and Radiofrequency Catheter Ablation

Intracardiac electrograms were recorded using a Prucka CardioLab Electrophysiology system (General Electric Medical Systems, Inc., Milwaukee, WI, United States). Three-dimensional electroanatomic mapping (NavX, St. Jude Medical, Inc., Minnetonka, MN, United States; CARTO, Biosense-Webster, Inc., Diamond Bar, CA, United States) was performed using a circumferential PV mapping catheter (Lasso, Biosense-Webster Inc.) through a long sheath (Schwartz left 1, St. Jude Medical, Inc.). Transseptal punctures were performed, and multiview pulmonary venograms were obtained. The details of the AFCA technique were described previously (5, 6). All patients underwent circumferential PV isolation (CPVI) during the *de novo* procedure. Two-thirds of the patients (62.1%) underwent the creation of a cavotricuspid isthmus block during the *de novo* procedure. Systemic anticoagulation was achieved with intravenous heparin to maintain an activated clotting time of 350–400 s during the procedure. After completion of the protocol-based ablation, the procedure was completed when no recurrence of AF was observed within 10 min after cardioversion with isoproterenol infusion (5–10 μg/min depending on β-blocker use, target sinus heart rate, 120 bpm) (6). Complications were defined according to the 2017 HRS (Heart Rhythm Society)/EHRA (European Heart Rhythm Association)/APHRS (Asia Pacific Heart Rhythm Society)/SOLAECE (Latin American Society of Cardiac Stimulation and Electrophysiology) expert consensus (7). Detailed definitions of the complications have been described previously (8).

### Follow-Up and Atrial Fibrillation Recurrence

We discharged patients not taking AADs except for those who had recurrent extra-PV triggers after the AFCA procedure, symptomatic frequent atrial premature beats, non-sustained atrial tachycardia, or an early recurrence of AF on telemetry during the admission period. Electrocardiography was performed for all patients visiting the outpatient clinic 1, 3, 6, and 12 months after AFCA and every 6 months thereafter or whenever symptoms developed. Twenty-four-hour Holter recordings were performed at 3, 6, and 12 months and every 6 months thereafter. Patients who reported episodes of palpitations suggestive of arrhythmia recurrence underwent Holter monitoring or event monitoring recordings.

The primary outcome was late recurrence defined as any episode of AF or atrial tachycardia (AT) lasting at least 30 s after a 3-month blanking period. Early recurrence was defined as any documentation of AF or AT recurrence on ECG within the 3-month blanking period. Follow-up lasted up to January 31, 2020 for the pre-COVID-19 era group and July 31, 2021, for the COVID-19 era group with equal follow-up durations for the groups with a minimum follow-up of 3 months (Supplementary Figure 1B).

### Statistical Analysis

Continuous variables are summarized as median (interquartile range), while categorical variables are summarized as frequencies (percentages). A Kaplan–Meier analysis with the log-rank test was used to calculate AF recurrence-free survival over time across groups. Multivariable Cox regression analysis was performed to estimate the relative hazards of AF recurrence. The following variables were adjusted: age, sex, duration of AF, body mass index, CHA_2_DS_2_-VASc, medical history, antiarrhythmic drug use, alcohol use, echocardiographic parameters, an inflammatory marker, ablation lesion set, and follow-up duration (variables in Table 1). The proportional hazards assumption was tested based on Schoenfeld residuals (9).

**TABLE 1:** Baseline characteristics of patients with paroxysmal and persistent atrial fibrillation undergoing catheter ablation.

Variables	Paroxysmal AF (*n* = 660)				Persistent AF (*n* = 394)			
	Overall (*n* = 660)	Pre COVID-19 era (*n* = 318)	COVID-19 era (*n* = 342)	*P*-value	Overall (*n* = 394)	Pre COVID-19 era (*n* = 135)	COVID-19 era (*n* = 259)	*P*-value
Age, years	60 (52–67)	59 (51–66)	61 (54–68)	0.018	61 (53–67)	58 (50–65)	61 (55–68)	0.001
Female, *n* (%)	179 (27.1)	84 (26.4)	95 (27.8)	0.760	77 (19.5)	27 (20.0)	50 (19.3)	0.975
AF duration, months	15 (7–36)	14 (7–36)	15 (7–36)	0.628	20 (9–48)	19 (9–47)	20 (9–48)	0.746
BMI, kg/m^2^	24.6 (22.9–26.7)	24.5 (22.8–26.6)	24.7 (23.1–26.7)	0.562	25.3 (23.3–27.4)	25.7 (23.7–27.5)	25.2 (23.0–27.2)	0.092
CHA_2_DS_2_-VASc score	1 (0–2)	1 (0–2)	1 (0–2)	0.140	2 (1–2.75)	2 (1–2)	2 (1–3)	0.019
Comorbidities, *n* (%)								
Heart failure	54 (8.2)	22 (6.9)	32 (9.4)	0.317	102 (25.9)	32 (23.7)	70 (27.0)	0.553
Hypertension	298 (45.2)	150 (47.2)	148 (43.3)	0.354	208 (52.8)	65 (48.1)	143 (55.2)	0.220
Diabetes mellitus	92 (13.9)	47 (14.8)	45 (13.2)	0.625	75 (19.0)	27 (20.0)	48 (18.5)	0.828
Stroke	53 (8.0)	19 (6.0)	34 (9.9)	0.084	37 (9.4)	10 (7.4)	27 (10.4)	0.428
TIA	6 (0.9)	4 (1.3)	2 (0.6)	0.617	1 (0.3)	0 (0.0)	1 (0.4)	1.000
Vascular disease	20 (3.0)	10 (3.1)	10 (2.9)	1.000	24 (6.1)	3 (2.2)	21 (8.1)	0.036
Current drinking, *n* (%)	158 (23.9)	93 (29.2)	65 (19.0)	0.003	121 (30.7)	46 (34.1)	75 (29.0)	0.352
Total alcohol intake per week in current drinkers, g	51.8 (17.7–148.9)	64.8 (19.4–155.4)	51.8 (15.5–103.6)	0.155	77.7 (17.7–207.2)	90.7 (24.2–207.2)	77.7 (17.8–155.4)	0.544
Drinking frequency per week	1.5 (0.9–2.5)	1.5 (1.0–3.0)	1.0 (0.8–2.0)	0.190	2.0 (1.0–3.0)	2.0 (1.0–3.5)	1.5 (1.0–3.0)	0.375
AAD use prior to the ablation, *n* (%)								
Class Ic	378 (57.3)	189 (59.4)	189 (55.3)	0.316	158 (40.1)	51 (37.8)	107 (41.3)	0.568
Class III	306 (46.4)	142 (44.7)	164 (48.0)	0.441	264 (67.0)	98 (72.6)	166 (64.1)	0.112
Echocardiographic parameters								
LA dimension, mm	39 (35–43)	38 (35–43)	39 (36–43)	0.331	43 (39–46)	44 (40–48)	43 (39–45)	0.011
LV ejection fraction,%	65 (61–69)	65 (61–69)	65 (62–69)	0.448	62 (57–66)	61 (56–65)	62 (58–67)	0.030
E/Em	9.1 (7.4–11.8)	9.0 (7.2–11.1)	9.3 (7.6–12.3)	0.133	9.0 (7.3–11.5)	8.4 (7.4–10.6)	9.4 (7.4–12.0)	0.009
hsCRP, mg/dL	0.60 (0.30–1.10)	0.70 (0.40–1.37)	0.50 (0.20–1.00)	< 0.001	0.70 (0.40–1.35)	0.80 (0.60–1.72)	0.60 (0.20–1.20)	< 0.001
CPVI, *n* (%)	660 (100.0)	318 (100.0)	342 (100.0)	1.000	394 (100.0)	135 (100.0)	259 (100.0)	1.000
CTI, *n* (%)	399 (60.5)	206 (64.8)	193 (56.4)	0.035	256 (65.0)	111 (82.2)	145 (56.0)	< 0.001
Follow-up duration, months	11 (6–15)	11 (7–15)	12 (6–15)	0.898	11 (6–15)	11 (7–15)	11 (6–14)	0.609

*Values are presented as median (interquartile range) or n (%).*

*AAD, antiarrhythmic drug; AF, atrial fibrillation; BMI, body mass index; CPVI, circumferential pulmonary vein isolation; CTI, cavotricuspid isthmus; E/Em, ratio of the peak mitral flow velocity of the early rapid filling to the early diastolic velocity of the mitral annulus; hsCRP, high sensitive C-reactive protein; LA, left atrium; LV, left ventricle; TIA, transient ischemic attack.*

A two-sided *P*-value of less than 0.05 was considered statistically significant. Statistical analyses were performed using R version 4.0.2 software (The R Foundation).^1^

## Results

**FIGURE 1:**
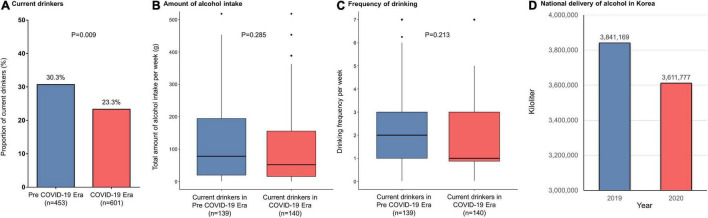
**(A)** Proportion of current drinkers, **(B)** total alcohol intake per week, **(C)** frequency of drinking per week in atrial fibrillation patients undergoing catheter ablation, and **(D)** amounts of national alcohol deliveries in Korea. COVID-19, coronavirus disease 2019.

**FIGURE 2:**
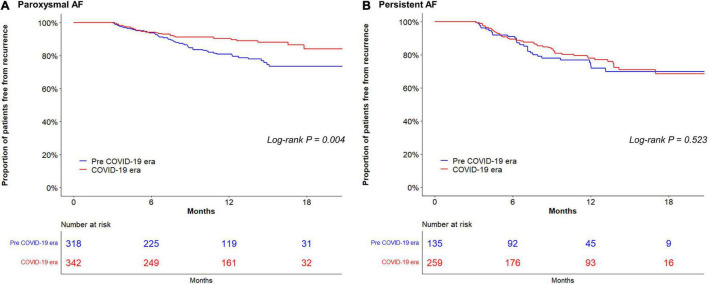
Kaplan–Meier analysis of atrial fibrillation: (AF) recurrence-free survival in paroxysmal AF **(A)**; and in persistent AF **(B)**. AF, atrial fibrillation; COVID-19, coronavirus disease 2019.

In the study population (24.3% women; median age, 60 years), 660 (62.6%) had paroxysmal AF. Patients ablated in the COVID-19 era tended to be older and more frequently had a history of stroke than those in the pre-COVID-19 era group (Table 1). The proportion of current drinkers was lower in the COVID-19 era than in the pre COVID-19 era (23.3% vs. 30.3%, respectively; *P* = 0.009) (Figure 1A) whereas there were no differences in the amount of weekly alcohol intake (Figure 1B) and the frequency of drinking (Figure 1C) between the groups. Procedural complication rates did not differ between the pre-COVID-19 era (2.4%) and COVID-19 era (3.2%) groups (*P* = 0.606). There were no differences in the compliances to Holter monitoring between the pre-COVID-19 era and COVID-19 era groups (Table 2). Among those with paroxysmal AF, the rate of late recurrence was significantly lower in those ablated in the COVID-19 era than in those ablated in the pre-COVID-19 era (9.4% vs. 17.0%, respectively; *P* = 0.005) during a median follow-up of 11 months (Table 2). The cumulative incidence of late recurrence at 1 year of follow-up was significantly lower in the COVID-19 era group (9.8%) than in the pre-COVID-19 era group (19.2%; log-rank *P* = 0.004) (Figure 2A). In multivariable Cox regression, the patients ablated in the COVID-19 era were at a lower risk of recurrence than those ablated in the pre-COVID-19 era [adjusted hazard ratio (HR) 0.56; 95% confidence interval (CI), 0.35–0.90]. In patients with persistent AF, the recurrence rate did not significantly differ between the pre-COVID-19 and COVID-19 eras (18.9% vs. 21.5%, respectively; *P* = 0.636) during the median follow-up of 11 months (Table 2). There was no difference in the cumulative incidence of recurrence at 1 year of follow-up between the two eras (26.4% in the pre-COVID-19 era vs. 22.1% in the COVID-19 era; log-rank *P* = 0.523) (Figure 2B). Risk of late recurrence did not differ between the two eras in multivariable Cox regression (adjusted HR 0.84; 95% CI, 0.47–1.53).

**TABLE 2:** Clinical rhythm outcomes.

	Paroxysmal AF (*n* = 660)			Persistent AF (*n* = 394)		
	Pre COVID-19 era	COVID-19 era	*P*-value	Pre COVID-19 era	COVID-19 era	*P*-value
	(*n* = 318)	(*n* = 342)		(*n* = 135)	(*n* = 259)	
Follow-up months	11 (7–15)	12 (6–15)	0.898	11 (7–15)	11 (6–14)	0.609
Compliant to Holter monitoring	235 (73.9)	235 (68.7)	0.166	90 (66.7)	145 (56.0)	0.052
Post-ablation medication						
ACEi, or ARB, *n* (%)	105 (33.0)	100 (29.2)	0.335	43 (31.9)	110 (42.5)	0.052
Beta blocker, *n* (%)	134 (42.1)	146 (42.7)	0.949	79 (58.5)	154 (59.5)	0.942
Statin, *n* (%)	115 (36.2)	144 (42.1)	0.138	53 (39.3)	123 (47.5)	0.146
AAD use						
AADs at discharge, *n* (%)	93 (29.2)	76 (22.2)	0.048	70 (51.9)	95 (36.7)	0.005
AADs after 3 months, *n* (%)	116 (36.5)	87 (27.0)	0.013	84 (62.2)	106 (45.1)	0.002
AADs at final follow-up, *n* (%)	92 (28.9)	80 (25.2)	0.326	65 (48.1)	109 (47.4)	0.975
Early recurrence, *n* (%)	80 (25.2)	58 (17.0)	0.013	73 (54.1)	129 (49.8)	0.485
Recurrence type AF, *n* (% in early recur)	72 (90.0)	54 (93.1)	0.739	68 (93.2)	123 (95.3)	0.735
Recurrence type AT, *n* (% in early recur)	8 (10.0)	4 (6.9)		5 (6.8)	6 (4.7)	
Late recurrence, *n* (%)	54 (17.0)	32 (9.4)	0.005	29 (21.5)	49 (18.9)	0.636
Recurrence type AF, *n* (% in recur)	48 (88.9)	29 (90.6)	1.000	28 (96.6)	48 (98.0)	1.000
Recurrence type AT, *n* (% in recur)	6 (11.1)	3 (9.4)		1 (3.4)	1 (2.0)	
Cardioversion, *n* (% in recur)	10 (18.5)	1 (3.1)	0.083	12 (41.4)	12 (25.0)	0.211

*AAD, antiarrhythmic drug; ACEi, angiotensin-converting enzyme inhibitor; AF, atrial fibrillation; ARB, angiotensin receptor blocker; AT, atrial tachycardia; COVID-19, coronavirus disease 2019.*

## Discussion

The reasons for the association of the pandemic situation and public restrictions with a lower recurrence rate after AFCA in paroxysmal AF patients are unclear. After the implementation of NPIs in Korea, the monthly drinking rate, indicating the proportion of citizens who drink at least once a month for the past year, decreased from 59.9% in 2019 to 54.7% in 2020, the lowest value in the last 15 years (10). The Korean nationwide liquor delivery decreased from 3,841,169 kl in 2019 to 3,611,777 kl in 2020 (Figure 1D) (11). Takahashi et al. reported that alcohol reduction was associated with a 37% lower risk of recurrence after AFCA (12). In particular, the risk almost halved in those with paroxysmal AF (12). The proportion of current drinkers among patients with paroxysmal AF in this study was significantly higher in the pre-COVID-19 era (29.2%) than in the COVID-19 era (19.0%; *P* = 0.003), whereas there was no significant difference between 34.1% in the pre-COVID-19 era and 29.0% in the COVID-19 era among patients with persistent AF (*P* = 0.352). High-sensitivity C-reactive protein levels were also lower among patients undergoing AFCA in the COVID-19 era than those in the pre-COVID-19 era. Importantly, up-regulation of inflammatory biomarkers has been shown to be a valid predictor for AF recurrence (13, 14), and inflammation is known to alter atrial electrophysiology and structure to increase vulnerability to AF (15). Thus, changes in alcohol habits and systemic inflammation during the period of COVID-19 pandemic and associated social distancing might partly explain the results of this study.

This retrospective observational cohort study was performed at a single center and included patients using strict selection criteria for AF ablation; hence, our findings cannot be used to establish causal relationships. Although the follow-up period of this study was designed to enable a 3-month blanking period in all patients and to equalize follow-up durations between groups, there might be a discrepancy depending on the timing at which procedures were performed during the inclusion period. However, there were no differences in the follow-up durations between patients in the pre-COVID-19 and COVID-19 groups. In the pandemic period, the Korean medical system was under normal operation, and all elective AFCA procedures proceeded in the same manner as that before the pandemic without significant delay. Among the patients included this study, there were no differences in the compliances to Holter monitoring between the pre-COVID-19 era and COVID-19 era groups. In both paroxysmal AF and persistent AF patients, the uses of AADs at discharge and at 3 months of follow-up were more frequently observed in the pre-COVID-19 era than in the COVID-19 era, whereas there were no differences at the time of final follow-up. This difference might impact the outcomes.

## Conclusion

This study reveals comparable outcomes of AFCA performed during the COVID-19 pandemic vs. the pre-pandemic period. Rather, a striking decrease in AF recurrence after catheter ablation was observed in patients with paroxysmal AF during the COVID-19 outbreak, whereas no change was observed in those with persistent AF.

## Data Availability Statement

The raw data supporting the conclusions of this article will be made available by the authors, without undue reservation.

## Ethics Statement

The studies involving human participants were reviewed and approved by the study protocol adhered to the principles of the Declaration of Helsinki and was approved by the Institutional Review Board at Yonsei University Health System. The patients/participants provided their written informed consent to participate in this study.

## Author Contributions

H-NP contributed to the conception and design of the work, acquisition of data, and critical revision of the manuscript. DK contributed to the conception and design of the work, interpretation of data, and drafting of the manuscript. HTY, T-HK, J-SU, BJ, and M-HL contributed to the conception and design of the work and acquisition data. H-NP attested that all listed authors meet authorship criteria and that no others meeting the criteria have been omitted. All authors approved the final version to be published and agreed to be accountable for all aspects of the work in ensuring that questions related to the accuracy or integrity of any part of the work are appropriately investigated and resolved.

## Conflict of Interest

The authors declare that the research was conducted in the absence of any commercial or financial relationships that could be construed as a potential conflict of interest.

## Publisher’s Note

All claims expressed in this article are solely those of the authors and do not necessarily represent those of their affiliated organizations, or those of the publisher, the editors and the reviewers. Any product that may be evaluated in this article, or claim that may be made by its manufacturer, is not guaranteed or endorsed by the publisher.

## Footnotes

1
www.R-project.org

## References

[1] World Health Organization [WHO]. *Coronavirus Disease (COVID-19) Situation Reports.* (2021). Available online at: https://covid19.who.int/WHO-COVID-19-global-data.csv (accessed October 23, 2021).

[2] Huh K Jung J Hong J Kim M Ahn JG Kim JH Impact of nonpharmaceutical interventions on the incidence of respiratory infections during the coronavirus disease 2019 (COVID-19) outbreak in Korea: a nationwide surveillance study. *Clin Infect Dis.* (2021) 72:e184–91. 10.1093/cid/ciaa1682 33150393 PMC7665442

[3] Lakkireddy DR Chung MK Gopinathannair R Patton KK Gluckman TJ Turagam M Guidance for cardiac electrophysiology during the COVID-19 pandemic from the Heart Rhythm Society COVID-19 Task Force; Electrophysiology Section of the American College of Cardiology; and the Electrocardiography and Arrhythmias Committee of the Council on Clinical Cardiology, American Heart Association. *Circulation.* (2020) 141:e823–31. 10.1161/CIRCULATIONAHA.120.047063 32228309 PMC7243667

[4] Nagueh SF Smiseth OA Appleton CP Byrd BF III Dokainish H Edvardsen T Recommendations for the evaluation of left ventricular diastolic function by echocardiography: an update from the American Society of Echocardiography and the European Association of Cardiovascular Imaging. *J Am Soc Echocardiogr.* (2016) 29:277–314. 10.1016/j.echo.2016.01.011 27037982

[5] Kim D Shim J Kim YG Yu HT Kim TH Uhm JS Malnutrition and risk of procedural complications in patients with atrial fibrillation undergoing catheter ablation. *Front Cardiovasc Med.* (2021) 8:736042. 10.3389/fcvm.2021.736042 PMC8572960 34760941

[6] Kim D Hwang T Kim M Yu HT Kim TH Uhm JS Extra-pulmonary vein triggers at de novo and the repeat atrial fibrillation catheter ablation. *Front Cardiovasc Med.* (2021) 8:759967. 10.3389/fcvm.2021.759967 34805314 PMC8600078

[7] Calkins H Hindricks G Cappato R Kim YH Saad EB Aguinaga L 2017 HRS/EHRA/ECAS/APHRS/SOLAECE expert consensus statement on catheter and surgical ablation of atrial fibrillation. *Europace.* (2018) 20:e1–160. 10.1093/europace/eux274 29016840 PMC5834122

[8] Lee JH Yu HT Kwon OS Han HJ Kim TH Uhm JS Atrial wall thickness and risk of hemopericardium in elderly women after catheter ablation for atrial fibrillation. *Circ Arrhythm Electrophysiol.* (2021) 14:e009368. 10.1161/CIRCEP.120.009368 33657832

[9] Grambsch PM Therneau TM. Proportional hazards tests and diagnostics based on weighted residuals. *Biometrika.* (1994) 81:515–26. 10.1093/biomet/81.3.515

[10] Korea Disease Control and Prevention Agency. *Community Health Survey Reports 2020.* (2021). Available online at: https://chs.kdca.go.kr/chs/stats/statsMain.do (accessed December 10, 2021).

[11] Statistics Korea. *E-National Index.* (2021). Available online at: https://www.index.go.kr/potal/main/EachDtlPageDetail.do?idx_cd=2824 (accessed December 10, 2021).

[12] Takahashi Y Nitta J Kobori A Sakamoto Y Nagata Y Tanimoto K Alcohol consumption reduction and clinical outcomes of catheter ablation for atrial fibrillation. *Circ Arrhythm Electrophysiol.* (2021) 14:e009770. 10.1161/CIRCEP.121.009770 33999699

[13] Korodi S Toganel R Benedek T Hodas R Chitu M Ratiu M Impact of inflammation-mediated myocardial fibrosis on the risk of recurrence after successful ablation of atrial fibrillation - the FIBRO-RISK study: protocol for a non-randomized clinical trial. *Medicine.* (2019) 98:e14504. 10.1097/MD.0000000000014504 30817568 PMC6831404

[14] Richter B Gwechenberger M Socas A Zorn G Albinni S Marx M Markers of oxidative stress after ablation of atrial fibrillation are associated with inflammation, delivered radiofrequency energy and early recurrence of atrial fibrillation. *Clin Res Cardiol.* (2012) 101:217–25. 10.1007/s00392-011-0383-3 22102100

[15] Zhou X Dudley SC Jr. Evidence for inflammation as a driver of atrial fibrillation. *Front Cardiovasc Med.* (2020) 7:62. 10.3389/fcvm.2020.00062 32411723 PMC7201086

