# Supplementary material for: Impact of the COVID-19 Pandemic and Public Restrictions on Outcomes After Catheter Ablation of Atrial Fibrillation
Source: Front Cardiovasc Med. 2022 Mar 24;9:836288. doi: 10.3389/fcvm.2022.836288 (PMC8987708; doi:10.3389/fcvm.2022.836288)
Supplement: Supplementary file 1 [file Data_Sheet_1.PDF]

Supplemental Figure 1. (A) Number of COVID-19 cases in Korea, (B) study inclusion criteria and follow-up periods, and (C) flowchart of the study.

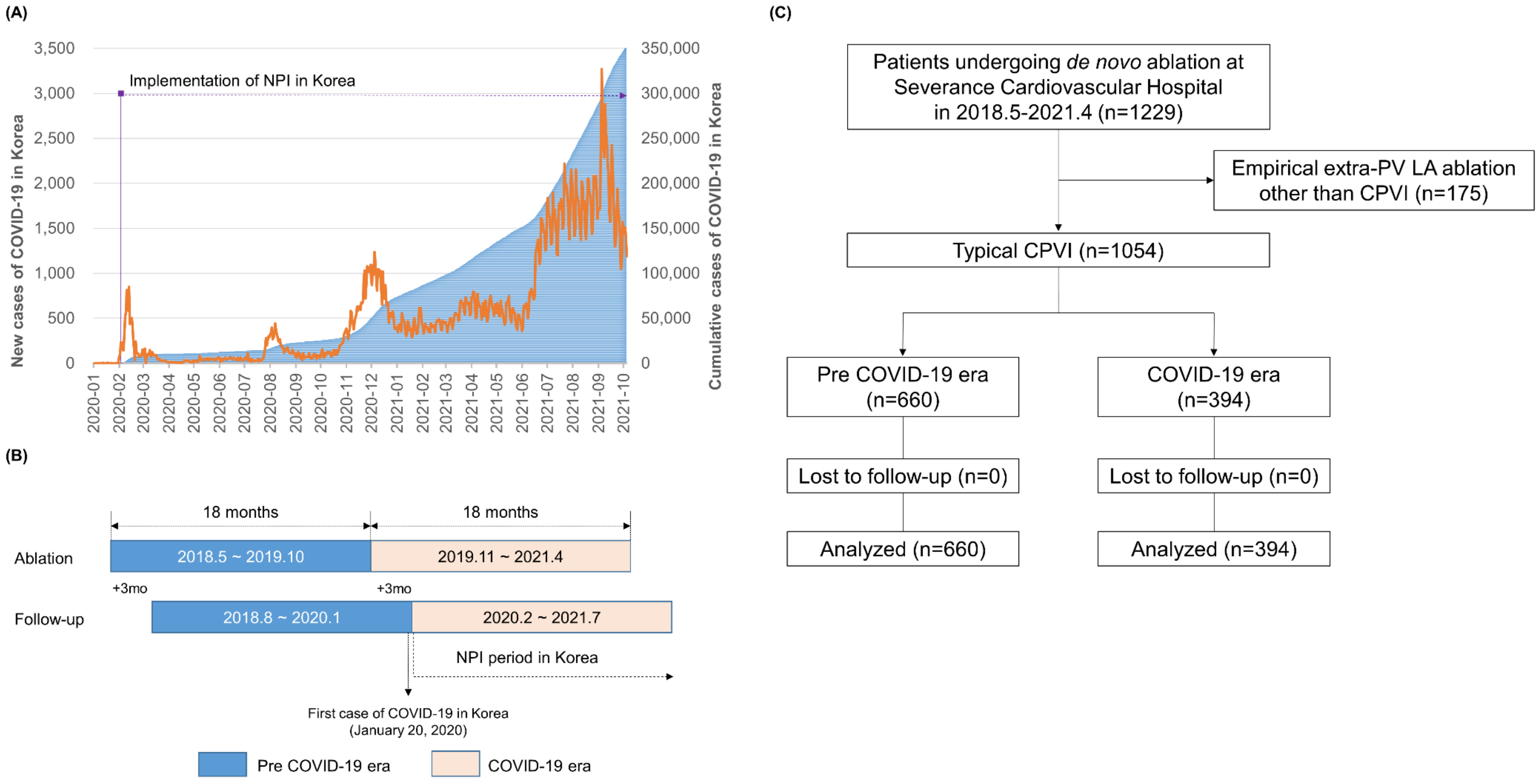

AF, atrial fibrillation; COVID-19, coronavirus disease 2019; CPVI, circumferential pulmonary vein isolation; NPI, nonpharmaceutical intervention; PV, pulmonary vein.
